# Supplementary material for: AffyMAPSDetector: a software tool to characterize Affymetrix GeneChip™ expression arrays with respect to SNPs
Source: BMC Bioinformatics. 2007 Jul 30;8:276. doi: 10.1186/1471-2105-8-276 (PMC1959249; doi:10.1186/1471-2105-8-276)
Supplement: Additional file 13 — AffyMAPSDetector v1 source code. AffyMAPSDetector source code. [file 1471-2105-8-276-S13.zip › AffyMAPSDetector_v1_src/doc/index-files/index-8.html]

H-Index


|  |  |  |  |
| --- | --- | --- | --- |
| **Overview** \| Package \| Class \| Use \| **Tree** \| **Deprecated** \| **Index** \| **Help** | | |  |
| **PREV LETTER**   **NEXT LETTER** | **FRAMES**    **NO FRAMES**     **All Classes** |  |  |


A B C D E F G H I J L M N O P R S T V W X Y 

---


## **H**

**HAS\_SNPS\_FILENAME** - Static variable in class affychip.snpdetector.Constant: **HEADER** - Static variable in class affychip.snpdetector.model.GeneInfo: **HEADER** - Static variable in class affychip.snpdetector.model.SnpInfo: **HEAT\_MAP** - Static variable in class affychip.snpdetector.Constant: **HELP\_I18N** - Static variable in class jwf.Wizard: **hasHelp()** - Method in class affychip.snpdetector.ui.StepOnePanel: Has this panel got help? Defaults to false, override to change. **hasHelp()** - Method in class jwf.WizardPanel: Has this panel got help? Defaults to false, override to change. **hasNext()** - Method in class affychip.snpdetector.ui.StepOnePanel: Is there be a next panel? **hasNext()** - Method in class affychip.snpdetector.ui.StepThreePanel: **hasNext()** - Method in class affychip.snpdetector.ui.StepTwoPanel: **hasNext()** - Method in class jwf.NullWizardPanel: Is there be a next panel? **hasNext()** - Method in class jwf.WizardPanel: Is there be a next panel? **hasSNPs()** - Method in class affychip.snpdetector.model.Probe: **help()** - Method in class jwf.WizardPanel: Override this method to provide help.

---


|  |  |  |  |
| --- | --- | --- | --- |
| **Overview** \| Package \| Class \| Use \| **Tree** \| **Deprecated** \| **Index** \| **Help** | | |  |
| **PREV LETTER**   **NEXT LETTER** | **FRAMES**    **NO FRAMES**     **All Classes** |  |  |


A B C D E F G H I J L M N O P R S T V W X Y 

---
